# Design of a Patient Voice App Experience for Heart Failure Management: Usability Study

**DOI:** 10.2196/41628

**Published:** 2022-12-06

**Authors:** Antonia Barbaric, Cosmin Munteanu, Heather Ross, Joseph A Cafazzo

**Affiliations:** 1 Centre for Digital Therapeutics Techna Institute University Health Network Toronto, ON Canada; 2 Institute of Health Policy, Management and Evaluation Dalla Lana School of Public Health University of Toronto Toronto, ON Canada; 3 Institute of Biomedical Engineering University of Toronto Toronto, ON Canada; 4 Institute for Communication, Culture, Information, and Technology University of Toronto Mississauga, ON Canada; 5 Technologies for Aging Gracefully Lab University of Toronto Toronto, ON Canada; 6 Ted Rogers Centre for Heart Research University Health Network Toronto, ON Canada; 7 Department of Medicine University of Toronto Toronto, ON Canada; 8 Peter Munk Cardiac Centre University Health Network Toronto, ON Canada; 9 Department of Computer Science University of Toronto Toronto, ON Canada; 10 Healthcare Human Factors Techna Institute University of Toronto Toronto, ON Canada

**Keywords:** heart failure, self-management, digital therapeutics, voice-activated technology, smart speaker, usability study, formative evaluation, mobile phone, smartphone

## Abstract

**Background:**

The use of digital therapeutics (DTx) in the prevention and management of medical conditions has increased through the years, with an estimated 44 million people using one as part of their treatment plan in 2021, nearly double the number from the previous year. DTx are commonly accessed through smartphone apps, but offering these treatments through additional platforms can improve the accessibility of these interventions. Voice apps are an emerging technology in the digital health field; not only do they have the potential to improve DTx adherence, but they can also create a better user experience for some user groups.

**Objective:**

This research aimed to identify the acceptability and feasibility of offering a voice app for a chronic disease self-management program. The objective of this project was to design, develop, and evaluate a voice app of an already-existing smartphone-based heart failure self-management program, *Medly*, to be used as a case study.

**Methods:**

A voice app version of *Medly* was designed and developed through a user-centered design process. We conducted a usability study and semistructured interviews with patients with heart failure (N=8) at the Peter Munk Cardiac Clinic in Toronto General Hospital to better understand the user experience. A *Medly* voice app prototype was built using a software development kit in tandem with a cloud computing platform and was verified and validated before the usability study. Data collection and analysis were guided by a mixed methods triangulation convergence design.

**Results:**

Common themes were identified in the results of the usability study, which involved 8 participants with heart failure. Almost all participants (7/8, 88%) were satisfied with the voice app and felt confident using it, although half of the participants (4/8, 50%) were unsure about using it in the future. Six main themes were identified: changes in physical behavior, preference between voice app and smartphone, importance of music during voice app interaction, lack of privacy concerns, desired reassurances during voice app interaction, and helpful aids during voice app interaction. These findings were triangulated with the quantitative data, and it concluded that the main area for improvement was related to the ease of use; design changes were then implemented to better improve the user experience.

**Conclusions:**

This work offered preliminary insight into the acceptability and feasibility of a *Medly* voice app. Given the recent emergence of voice apps in health care, we believe that this research offered invaluable insight into successfully deploying DTx for chronic disease self-management using this technology.

## Introduction

### Background

The prevalence of heart failure (HF) continues to be on the rise as more people are surviving cardiovascular disease [1]. Cardiovascular diseases can cause the heart muscle to become damaged and weak, leading to the development of HF. HF occurs when the pumping action of the heart muscle is not strong enough to meet the needs of the body or when the heart muscle does not relax properly to accommodate blood flow back into the heart. When this occurs, fluid can build up in the lungs and other parts of the body, such as the ankles, creating congestion in the lungs, which results in a lack of oxygen being delivered to the rest of the body [2]. As of 2017, it is estimated that 64.3 million people are living with HF globally [3], and many countries are reporting a steady increase in this condition’s prevalence [1].

HF creates a burden not only on health care resources and expenditures [1] but also on the patient’s well-being if not cared for properly [4]. HF limits a patient’s capacity to live well through physical, psychological, or social means [5]. Patient self-management plays an integral role in the treatment of HF, with studies reporting improved health outcomes, decreased clinic visits, and decreased health costs [6]. Mobile health (mHealth) is a type of digital health technology that involves the use of mobile devices (smartphone, patient monitoring device, wireless devices, etc) for medical and public health practices [7] and enables the integration of self-care support into a patient’s daily routine [8]. Smartphone apps are one of the most popular tools for helping patients diagnosed with chronic conditions manage their health at home [9].

*Medly* is an evidence-based, HF self-management program that has been developed by the University Health Network (UHN) and is implemented as part of the standard of care at UHN’s Peter Munk Cardiac Centre (PMCC) [10]. This program is available to patients as a prescribed digital therapeutic, cleared by Health Canada in 2020 [11]. *Medly* consists of a smartphone app that enables patients to log clinically relevant physiological measurements and symptoms daily, which is then used in the *Medly* algorithm to generate an automated self-care message. The care team is able to review the patient’s data daily and can also view current trends and historic data. If the algorithm detects that the patient’s health is deteriorating, the care team receives alerts via email and on the dashboard and is able to contact the patient to provide advice. Previous studies evaluating *Medly* have proven that this program can reduce health service use and improve clinical, quality of life, and patient self-care outcomes [10].

Given these encouraging results, a longitudinal study measuring adherence was also performed on the *Medly* program. The data gathered over 12 months concluded that adherence to the program was inconsistent among all age groups and kept declining as time went on [12]. Furthermore, other studies have shown that various patient demographics struggle to use mobile technologies, such as touch screen devices and smartphones, specifically older adults and those with cognitive and physical impairments [13-19]. Although mHealth remains to be one of the most popular platforms for chronic disease self-management programs [9], there are still some patient demographics who struggle to interact with this technology. Voice apps have the potential to improve the user interaction of programs such as *Medly* based on emerging evidence of this technology being used in the self-management of chronic diseases. This study was performed and designed as the first step in determining whether it would be feasible and beneficial to create, deploy, and use a voice app of *Medly*. If results show promise, future studies can go beyond feasibility to then investigate implementation and whether certain demographics benefit more from this technology than others.

Voice apps are an emerging technology in the health care field, ranging from integration with in-clinic registration processes [20] to helping people manage their chronic illness and live independently in their homes [21-25]. There is growing interest in smart speakers such as Google Home (Google LLC) and Amazon Alexa (Amazon Inc) because of their simple setup, ease of use, and low cost. Voice apps are deployed through voice user interfaces (VUIs) and enable ubiquitous connectivity by allowing the user to access services using their speech, making for a more convenient experience. VUI is the underlying software that processes and handles speech inputs to allow the user to interact with various apps. A wide range of devices now includes VUIs, such as smartphones and smart speakers. Research relating to voice apps for chronic illness is still in the development and piloting phases and has limited efficacy in testing to support final outcomes and conclusions [26]. As described by Sezgin et al [24,26], most voice apps currently being developed provide information and assistance services, which include general educational content and guidance, reminders, and tracking. There is limited research showcasing voice apps as a tool to provide more personalized, user-specific support, creating an opportunity to further investigate whether this technology can be used to provide health care services and support.

### Objectives

Guided by the findings from the literature review and coupled with a user-centered design (UCD) process, we sought to design, develop, and evaluate a voice app for the smartphone-based HF self-management program *Medly* to determine whether a voice app version adds benefit to the program’s current model of interaction and care. The *Medly* voice app was evaluated in a usability study, and the findings from this evaluation helped inform the final design and development of the *Medly* voice app, as presented in this paper.

## Methods

### Overview

The *Medly* voice app was created in two main phases: (1) design and development and (2) usability evaluation of the preliminary design. A prototype version of the *Medly* voice app was created and used in a usability study. Findings from the usability evaluation helped inform the redesign and redevelopment work needed to create a voice app that better met the needs of patients with HF. This section begins with an outline of the design and development specifications created for the *Medly* voice app, followed by the verification and validation protocols. The methods pertaining to the usability study design and data collection and analysis will then be explained.

The *Medly* voice app was designed and developed for deployment on VUIs and was built using an external cloud computing service. A mixed methods, triangulation convergence model was used to interpret the results. Both quantitative and qualitative data regarding the user’s experience when interacting with the *Medly* voice app prototype were collected and analyzed separately. Following the analysis of each data set, the results were converged for interpretation to identify the main conclusions. The findings from this usability study influenced the redesign and redevelopment work that occurred after this evaluation.

### Ethical Considerations

The usability study was performed in Toronto General Hospital, part of UHN, and was approved by UHN Research Ethics Board (19-5051.2). Interested participants were asked to sign a written consent form before being enrolled in the study. They were reminded that (1) they can contact the study coordinator at any point should they have questions, (2) their decision to participate or not has no impact on their clinical care in any way, and (3) they are free to withdraw from the study at any point with no impact on their clinical care. All information collected pertaining to observations, semistructured interviews, and questionnaire responses was identified by study number only. Neither participants’ name nor any identifying information will be used in any publication or presentation. The participants received a CAD $25 (US $18.34) Visa gift card for participating in the study.

### Design Process

The first phase of the UCD process, identifying user needs and ideation of the *Medly* voice app, was guided by a literature review, a market scan, and the existing *Medly* program requirements. Findings from the literature review and market scan revealed that many chronic disease self-management voice apps (not limited to just HF) used Amazon Alexa or Google Home devices for deployment. All the research studies shared the same purpose of identifying the feasibility and acceptability of using a voice app for the self-management of a specific chronic disease. The findings were preliminary but encouraging with high user satisfaction and usability, indicating that devices such as Amazon Alexa and Google Home are reliable to be used for self-management purposes. The design of the voice apps described in both the literature review and market scan revealed two useful findings: (1) the voice app conversation is designed to be short and succinct, and (2) some of the voice apps are deployed on devices that have large screens, meant to complement the interaction. These results encouraged the *Medly* voice app’s conversational design to be concise and to explore the possibility of using a screen to complement the conversational experience.

**Figure 1:**
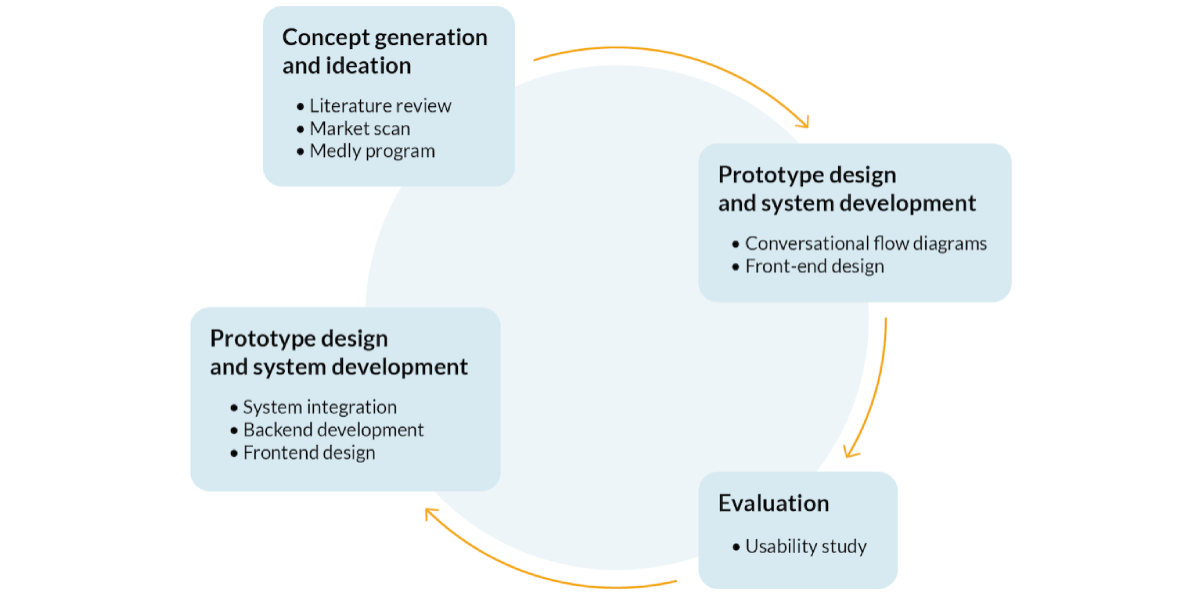
The design process was guided by a user-centered design process; details describing what was done during each phase are included.

The second phase of the UCD process involved using the information gathered in the first phase to develop and deploy a prototype of the *Medly* voice app on a VUI. User feedback about the *Medly* voice app was received through a usability study, performed with patients with HF in The Ted Rogers and Family Centre of Excellence in Heart Function clinic at the PMCC in Toronto General Hospital. On the basis of the usability study results, the prototype was further developed and redesigned to better meet the identified user needs and is expected to be evaluated in a future clinical study. Figure 1 illustrates the process that was followed for this project.

### Design Specifications of the Medly Voice App

Design requirements were created before developing the *Medly* voice app to help guide the development process and define the voice app’s functionalities. The main requirements implemented for the voice app are presented in Table S1 in Multimedia Appendix 1, which summarize the main objectives of the program. The voice app was designed to have the same functionalities as those of the mobile app, which include the following: (1) asking the user to measure their weight, blood pressure, and heart rate; (2) saving and storing those values in the *Medly* clinical dashboard; (3) asking the user a series of yes or no questions relating to HF symptoms; (4) processing the data using the *Medly* algorithm; and (5) outputting a message to the user based on the algorithm result. These requirements helped create an app that was appropriate for voice interaction and were based on research as well as guidelines related to VUI design [27,28]. Conversational flow diagrams were also created using VUI design guidelines, and each scenario was tested on a VUI following its implementation [29,30].

### Development Specifications of the Medly Voice App

The architecture of the *Medly* voice program consists of various components required to receive, store, and send data. A visual of the architectural design along with a description of how it operates is presented in Figure S1 in Multimedia Appendix 1.

Voice apps consist of both a VUI and app logic. When the user speaks, the VUI invokes the voice app, and a request is created and processed in the context of the voice app’s interaction model using tools such as machine learning, natural language understanding, and automatic speech recognition. Once the interaction model has processed the speech, a request is sent to the app logic, which provides a response back to the user. For the purposes of this project, an Alexa-hosted Skill built using the Amazon Alexa Skills Kit [31] processed and handled user requests and implemented the *Medly* voice app logic, but other frameworks could have been used as well.

Finally, the verification and validation of the *Medly* voice app were ongoing processes that were completed at various steps in the design process. Verification was done on the *Medly* voice app using a demonstration method to ensure that it met the design requirements and performed as expected. Different scenarios with various inputs were created to ensure that the produced results were as expected. Different conversational flows were brainstormed and used to help validate the voice app. These conversations were then tested using a web-based voice app simulator, which responds with voice and text and also includes the JavaScript Object Notation request to help fix errors.

### Usability Study

#### Overview

The purpose of the usability study was to investigate the potential of VUI devices as an option for patients to access the *Medly* program. Patients with HF were recruited and asked to explore the intuitiveness of the *Medly* voice app. The usability study consisted of 3 main components, which each patient completed: a usability session, the standardized System Usability Scale (SUS) questionnaire (Multimedia Appendix 2), and a qualitative semistructured interview session. The SUS questionnaire, developed in 1986, is a validated and reliable tool created to measure usability. The survey consists of 10 items and evaluates various products and services; it is commonly used in health care when implementing newer technologies [32]. The findings from this study helped identify the acceptability and ease of use of the voice app and also informed the service design that would be most appropriate to be implemented for a voice app offering a self-management program.

#### Participant Recruitment

A total of 8 participants were recruited for this study, and all of them used *Medly* as part of their standard of care. All the participants recruited were adults living with chronic HF and could read, write, and understand English. Patients using *Medley* were asked to participate so that unique insights and themes could be identified, as they already had experience with using the program. The participants were recruited through the *Medly* nurse coordinator, who provided a brief overview of the study to patients and then asked whether they were interested in participating before introducing them to the study coordinator. Most usability studies require 5 to 8 participants to receive response saturation [33,34]; therefore, we chose to recruit 8 participants to help identify the potential this platform may have as a health care delivery technology in the future, by identifying major themes emerging from the study.

#### Usability Study Design

Participants who volunteered attended a single, 1-hour session with the study coordinator after their scheduled appointment and were asked to complete four main tasks: (1) inputting measurements on the *Medly* smartphone app, (2) inputting measurements on the *Medly* voice app, (3) completing the SUS questionnaire, and (4) engaging in a semistructured interview with the study coordinator. Following this, the participants were then asked to verbally interact with the *Medly* voice app, with the help of an instruction card, using Amazon Alexa, which was also provided by the study coordinator. Observational notes were recorded throughout the session. The participants then completed a standardized, poststudy SUS questionnaire regarding their experience with the voice app. Once the participants completed the questionnaire, the study coordinator led individual semistructured interviews, which included questions pertaining to their perceptions of the usability of the *Medly* voice app and specific issues or areas for improvement. The results of this research highlighted key findings regarding the acceptability of integrating VUI platforms into patient self-care tools and informed decisions for future design iterations.

#### Data Collection and Analysis

Both quantitative and qualitative data were gathered during the usability study and triangulated when identifying the main conclusions from the results. The SUS questionnaire was analyzed as per standard protocol [32], and the results are graphically represented in the “Usability Study Findings” section below. The data were interpreted in conjunction with the qualitative interview findings and observational notes. An inductive, qualitative descriptive approach was used to analyze the interview responses and field notes; all transcripts were analyzed and coded by the study coordinator (AB), using Microsoft Word (Microsoft Corp). Because the purpose of the usability study was to collect user insights, it was not designed to be statistically powered; therefore, specific patient demographic characteristics such as age, ethnicity, etc were not collected or used in the analysis.

The mixed methods triangulation design followed a convergence model, with a larger emphasis being placed on the qualitative results. The findings from the SUS questionnaire were used to validate the main conclusions gathered from the interviews and field notes. The rationale for this approach was due to the usability study design; because a small sample size was needed to reach saturation, the interpretations from the quantitative data were used to support the qualitative findings.

## Results

### Preliminary Design of the Medly Voice App

A voice app prototype of *Medly* was created for the purpose of being used during a usability study to identify problems in the design, uncover any opportunities for improvement, and learn more about the users’ behaviors and preferences when interacting with the app. An instruction card was created and used to help guide the user through their interaction with the *Medly* voice app (Figure S2 in Multimedia Appendix 1). A preliminary design of the card was used by the participants in the study so that feedback could be collected about the design to make changes, if required.

**Figure 2:**
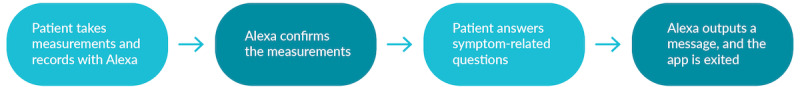
A high-level description of the conversational flow for the Medly voice app. “Milestones” are outlined and signify an important event.

A high-level overview of the conversation was first mapped out and created (Figure 2). Using this, the full conversation was designed and is described in more detail in Figure S3 in Multimedia Appendix 1. The first half of the voice app interaction consisted of the user measuring and recording their weight, blood pressure, and heart rate with nonlyrical music playing in the background in between prompts. Playing music in the background not only created a more pleasant user experience but was also a strategy used to ensure that the app did not time out [35]. After the user inputted their heart rate, the voice app reiterated the measurements it captured to the user and gave them an opportunity to correct any wrong measurements. Once the user recorded their measurements, the app began to ask them a series of yes or no questions regarding HF-related symptoms. Once this was completed, the app outputted a message and exited. The *Medly* algorithm was not implemented for the prototype because it was not needed to accomplish the purpose of the usability study.

An interaction model was then created to implement the conversational flow described earlier, and it consisted of intents, training phrases, and slot types. An intent was created for each piece of required information: weight, blood pressure, heart rate, and symptom-related questions. Within each intent, training phrases were added and consisted of predictable utterances the user may say to record their responses. Within each training phrase, the most important pieces of data were identified and labeled as a slot type (weight, blood pressure, and heart rate were categorized as *numbers*, and symptom responses were labeled as “yes” or “no”). The slot types were used to help extract the measurements needed to be sent to the *Medly* voice server. The measurements were extracted from the interaction model, and the app logic was used to help direct the flow of conversation. Once the required measurements were captured, a POST request was made to the *Medly* server, and a self-care message was sent back to the voice app to be relayed to the user.

### Usability Study Findings

#### SUS Results

**Figure 3:**
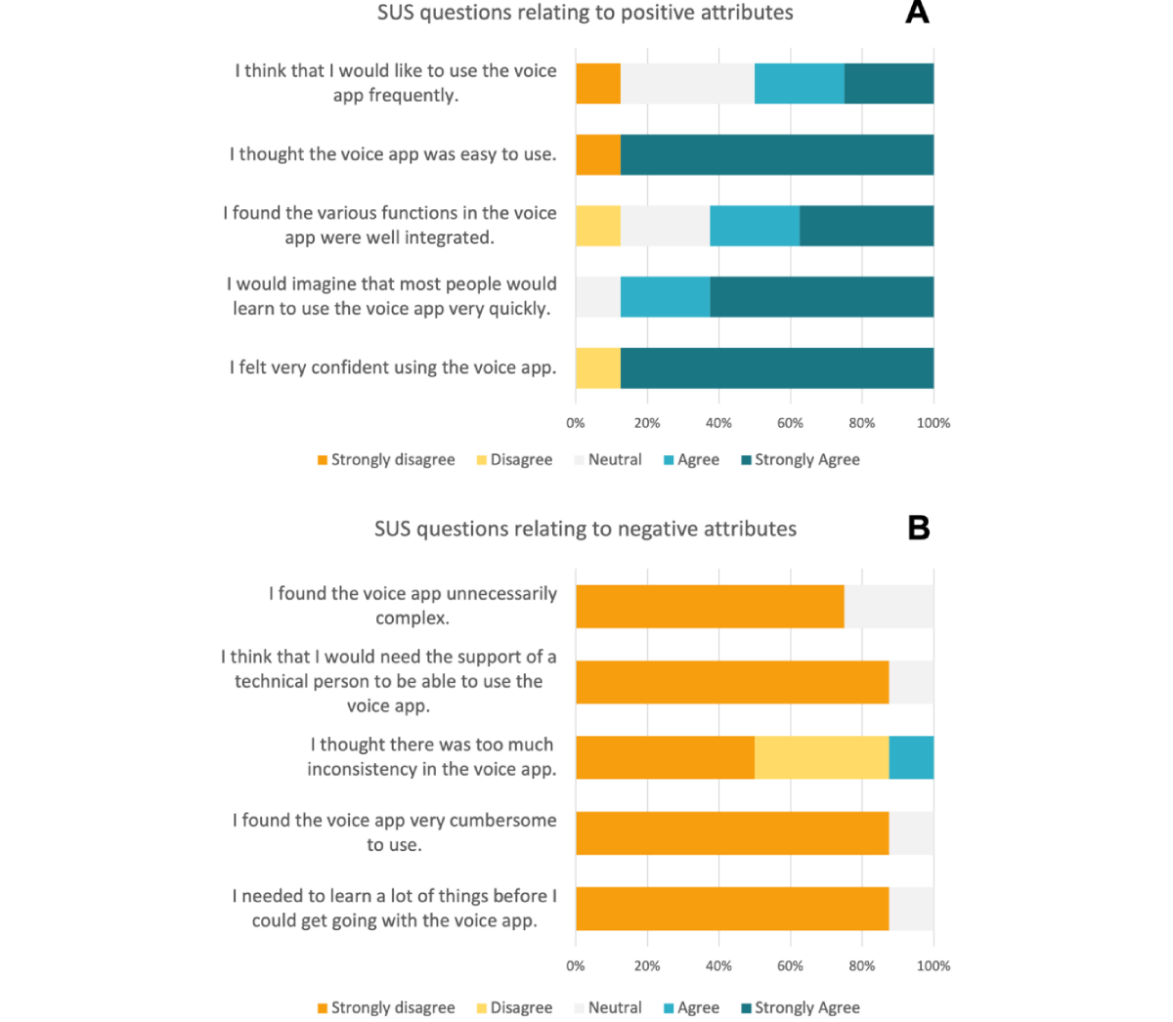
(A) Data showcasing the participant results for the questions in the System Usability Scale (SUS) relating to positive attributes of the Medly voice app system. (B) Data showcasing the participant results for the questions in the SUS relating to the negative attributes of the Medly voice app system.

A usability study was performed with 8 participants with HF from UHN’s Ted Rogers Center of Excellence for Heart Failure clinic. Saturation was successfully achieved with the sample size because of the common themes that emerged across the 8 sessions. After interacting with the voice app, the participants filled out the SUS questionnaire to describe their experience. The average SUS score was 92, out of 100 (SD 5.2), ranking the voice app in the 98th percentile based on previous studies. According to these data, most participants were satisfied with the design and development of the voice app. Results for the positive and negative statements of the SUS questionnaire are shown in Figures 3A and 3B, respectively.

Looking at the positive attribute statements, almost all participants (7/8, 88%) felt confident using the app and thought it was easy to use. This was further supported by the responses to the negative attribute statements, with the most popular response being “strongly disagree.” However, when asked whether they would use the voice app frequently, there was a greater divide in opinion, with the most popular response (3/8, 38%) being neutral.

#### SUS Triangulation With Qualitative Data

Overall, these findings showed promise when determining user acceptability because of the overall positive responses from the SUS questionnaire. Triangulating these results with the field notes and interview findings indicated areas for improvement regarding the ease of use. Although most of the responses were “strongly agree” or “strongly disagree,” 3 of the 10 statements had more variation: “I think that I would like to use the voice app frequently,” “I found the various functions in the voice app were well integrated,” and “I thought there was too much inconsistency in the voice app.” This can potentially be explained using 2 observations that were commonly seen during the user sessions. First, the voice app had difficulties understanding measurements when users said each digit separately for their weight, blood pressure, and heart rate (Table S1 in Multimedia Appendix 3). Second, the voice app would often time out whenever there was a small pause in between the prompt provided and the user’s response. In this scenario, it became difficult for the user to converse with the voice app. Given these observations (and their validation through questionnaire responses), some changes have been made to improve the patient experience.

#### Qualitative Findings

The observational notes and semistructured interview responses were analyzed to (1) identify and describe common themes and (2) create a list of potential errors that could occur while the user is interacting with the app. Both these deliverables provided insights and helped shape the redesign and redevelopment of the voice app. Six main themes were identified: (1) changes in physical behavior, (2) preference between voice app and smartphone, (3) importance of music during voice app interaction, (4) lack of privacy concerns, (5) desired reassurances during voice app interaction, and (6) helpful aids during voice app interaction.

One of the more notable themes was the observed change in behavior when the participants interacted with the voice app; most participants showed signs of nervousness and looked tense. However, as the conversation progressed, there was a noticeable difference in their demeanor, as they became more relaxed once they better understood the conversational flow. Some voice app features helped the users navigate through their session, making for a more pleasant experience, such as having music play in the background so that they did not feel rushed through the interaction and having the voice app reiterate measurements back to them to ensure that the device correctly recorded the values. Because the users were accustomed to seeing visual feedback of their measurements on their smartphones, they preferred having some type of feedback (verbal or visual) to ensure that the voice app heard them correctly. The participants heavily relied on the instruction card as an aid, and those who followed it closely were the most successful in completing the interaction. The use of music, reiteration feature, and instruction card was observed to be helpful during the interactions, and this was confirmed with the participants during the interview sessions. Insights were also provided as to why some patients found the smartphone device difficult to use, and the responses typically revolved around the idea of convenience. Some participants (4/8, 50%) found that inputting their data through a conversation (voice app) was easier for them than using their smartphone device. One of the participants said that they use their smartphone only for the Medly app and otherwise have it turned off. Finally, concerns about privacy were brought up only by 25% (2/8) of participants, indicating it may not be a prominent barrier to use as originally predicted. Further details describing these themes are outlined in Table S2 in Multimedia Appendix 3.

As mentioned previously, a list of potential risks, related to the interaction of the user with the voice app, was identified. The severity of each risk was assessed based on its likelihood and consequence. Mitigation strategies for each risk were developed and implemented in the voice app design during the redevelopment work. These data can be seen in Table S1 in Multimedia Appendix 3.

### Redevelopment of the Medly Voice App

#### Overview

The prototype built for the usability study was further developed to meet all the design requirements of the *Medly* voice app. The VUI device was also changed to incorporate screen and touch screen capabilities; this decision was based on the findings from the usability study. We expected that the touch screen will not be suitable for all patients; however, we were also led to believe (based on user feedback) that some may prefer it over the smartphone and screenless VUI device, especially for visual feedback.

#### Screen Design

**Figure 4:**
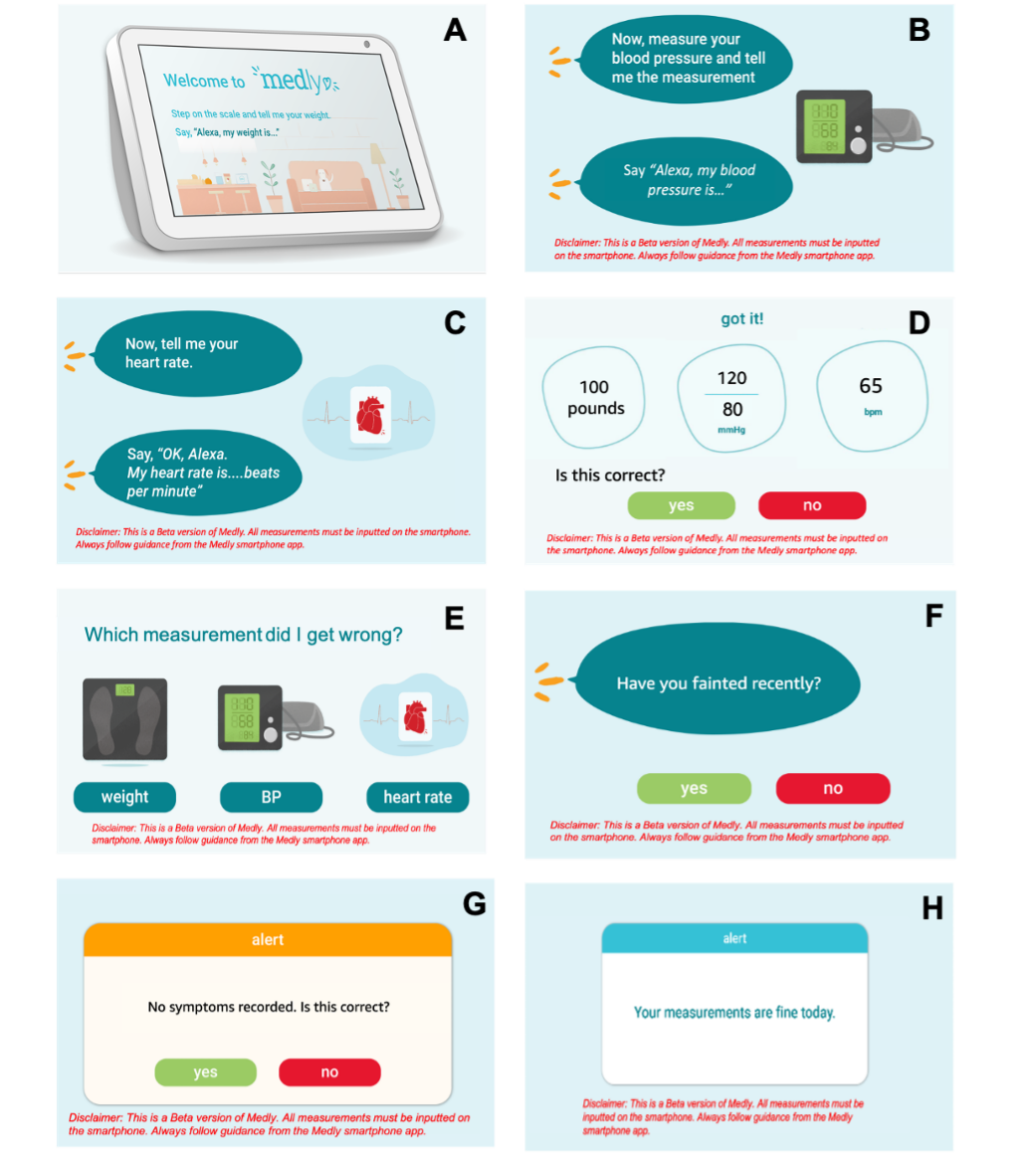
Screen designs showcased through images A-H, representing different events that occur in the conversation.

Having a screen display on the device meant that the users would be able to visually see instructions and measurements and also have the option of responding to yes or no questions using the touch screen. Although some demographics struggle to use touch screen, these interaction features are optional for the user. Although we predicted that the visual feedback will be helpful for most users and that having a bigger screen will be easier than the smartphone, the users will still be able to interact with the app using only their voice if they find it to be the easiest and simplest mode of interaction for them. Each screen was designed to only include the information the user needed to avoid any confusion. A consistent design theme, similar to the one used in the *Medly* smartphone app, was used for each screen display using the same color scheme, layout, and font size and style. The written cues on the screen aligned with the verbal prompts to avoid user confusion. Design guidelines were used for font size and layout to ensure that the prompts are presented at an appropriate reading size [35]. The screen design at various points of the conversation is shown in Figure 4.

The incorporation of a screen display for the *Medly* voice app also allows users to see their previous measurements and data trends, similar to what they were used to seeing when using the smartphone app. Although the implementation of seeing data history was not in scope for this project, it is a feature that is common in most mHealth apps, as it helps users understand their health status better.

#### Conversational Design

**Figure 5:**
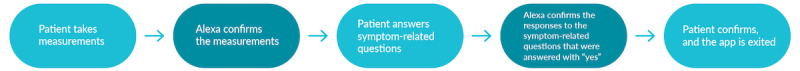
A high-level description of the conversational flow for the final version of the Medly voice app. A new milestone has been added to describe the Medly voice app, which confirms the responses to the symptom-related questions that were answered with “yes.”.

The conversational script used in the preliminary design stayed the same during the redevelopment work. Findings from the usability study helped inform which parts of the conversation were most prone to error and needed to be changed. More training phrases and symptom confirmations were new additions to the conversation, and the summary of the interaction is shown in Figure 5. A more detailed description of the new additions to the conversation can be seen in Figure S4 in Multimedia Appendix 1. With these new changes, the app only confirms the symptoms the user answered yes to for a more efficient process by giving them an opportunity to correct any wrong responses recorded. Once all the responses have been corrected, the data are sent to the *Medly* voice server. The voice server retrieves patient thresholds, sends the daily data to the *Medly* research server, and uses the *Medly* algorithm to send an output message back to the voice app, informing the patient on the status of their health.

#### Backend Development

The software architecture and *Medly* algorithm were implemented during the redevelopment phase of the project. Each output message generated to the user was a personalized response based on their measurements and preexisting thresholds. To receive data from the voice app and pull relevant patient thresholds, account linking was required to be set up to connect the patient’s identity from 2 different services. Personalized *Medly* accounts were created for each study participant and connected to their respective Amazon account. The *Medly* algorithm was built and deployed on the *Medly* voice server; it pulls data from the *Medly* app server as well as data sent from the VUI to generate an output message.

## Discussion

### Principal Findings

This manuscript describes the UCD approach that was used to develop a voice app experience for HF management. Accessing chronic disease management programs through voice apps has the potential to increase the uptake of these programs by making them more accessible, thereby reaching more patients. The evaluation of the voice app demonstrated user acceptability through the SUS questionnaire (92/100 average score), and the qualitative findings provided assurance of deployment feasibility, as the users were able to successfully interact with the voice app. Common themes from the usability sessions (preference for music playing during interaction, providing oral or visual feedback, and the instruction card being a helpful aid) served as feedback for the redevelopment phase of the project.

The impact of the findings from this study is promising, especially given the near-ubiquitous nature of smart speakers, such as Amazon Alexa and Google Home. These devices can be easily integrated within households, and they make it easy and convenient to perform tasks through simple conversations and offer flexibility to do multiple tasks simultaneously. Patients with chronic illness experience a constant need to record and transmit data to better track their health, and voice apps, deployed through smart speakers, can help them accomplish this seamlessly, not only because it uses speech to collect these data but also because it can guide the user through their tasks more explicitly than the smartphone.

As this platform requires less technical knowledge than the smartphone (mHealth apps), those who struggle to use technology have the opportunity to participate in the program. According to the usability study observations, most participants felt that it was easy to interact with the voice app because it only required conversation and no technical background. The platform’s multilingual capability offers another opportunity, which may make some more individuals willing to use it to record measurements. VUIs also allow users to initiate a call to anyone on their list through voice command; this can be useful in cases where they need assistance but cannot reach their phone to call for help. This scenario often occurs with older adults, who are also the most common group to be diagnosed with chronic illnesses and, as a result, are prescribed digital therapeutics.

It is also important to consider the implications of using voice apps for chronic disease self-management. When inputting measurements on an mHealth app, it is typical for the user to see their data on the screen before submitting. With a VUI that solely relies on verbal communication, visual feedback is no longer a possibility. The participants commented on the importance of knowing which measurements were recorded on the voice app and valued the ability to see historical data and trends on the smartphone app. With these findings, we incorporated verbal feedback as a necessary component in the conversational design to help with user confidence. Adding this step in the conversation comes at the cost of prolonging the time it takes to complete the interaction but is not necessarily a drawback because of the lesser focus required when compared with using a smartphone.

Although users only need to use speech when interacting with VUIs, the conversation style may vary significantly when compared with human-to-human interaction. During this study, the users who had never used these types of devices before felt the need to raise their voice in hopes that it heard and understood them better. Expelling this energy repeatedly may make the user feel tired, as one of the participants noted, especially because the demographic that uses these digital therapeutics are older adults. Without knowing how the voice app will respond, a difference in body language was also observed, namely users tensed up, felt the need to sit up straighter, and focused more when interacting with the device. This behavior is in stark contrast to how users interacted with the smartphone, as their physical body language while using the smartphone indicated a much more relaxed behavior. Despite these differences, it is expected that user body language will shift toward a more relaxed behavior as they become more comfortable with using voice apps.

Finally, it is also important to consider the potential barriers to entry that exist as a result of using this technology. For example, most VUI devices require the user to have an account to interact with the device, creating an accessibility issue. Another barrier to entry is the requirement to have a constant, reliable internet connection to use the device. This is in contrast to the *Medly* smartphone app which can be accessed offline and is mobile. Not only can this create an equity issue for those who do not have internet, but it can also be problematic when internet issues and power outages occur.

### Comparison With Prior Work

To our knowledge, this study is one of the first to investigate the acceptability and feasibility of a personalized chronic disease self-management voice app, designed to be used by patients with HF in their homes. Our work is similar to other recent studies that have used either an Amazon Alexa or a Google Home device to deploy a self-management voice app for various chronic diseases, including type 2 diabetes [23]. The usability study provided by Cheng et al [23] collected quantitative data through a survey, asking participants whether they preferred a smartphone or voice app; similar to the findings in our study, 8 out of 10 participants preferred the voice app. In our study, qualitative data were used to further explore the high acceptability response. Voice apps have also been developed for heart disease but for purposes other than self-management [21,22]; Jadczyk et al [20] developed one for patients to interact with during in-clinic registration to streamline the process. The usability study performed was in a controlled laboratory environment but revealed 97.5% accuracy when converting speech to text. In summary, our findings are consistent with the existing literature, which provide preliminary assessments of voice apps, an emerging technology in health care. Using a systematic approach, we were able to gather more insights about the feasibility and acceptability of voice apps through quantitative and qualitative data collection.

### Limitations

Although the results from this research project showed promise for future use, it is important to acknowledge the limitations associated with this work.

The main limitation of usability studies is the short interaction time. Although the observations and feedback from the participants were helpful in identifying the potential that voice apps may have for chronic disease management, they only interacted with the technology for a short period. Future work should include a study that requires the users to interact with the device for a longer period to give them more experience with the voice app to reflect more grounded feedback.

A sample size of 8 participants was chosen because the usability study was only focused on gathering user insights and not statistically relevant data. According to the literature, the total participants needed for a usability study is dependent on when saturation is achieved in results. In most cases, this occurs when the sample size is typically between 5 and 8 participants [36]. Therefore, it is recommended that multiple usability tests occur with fewer users (between 5 and 8 participants) and that changes be made between usability tests to mitigate challenges observed in previous sessions. The research showcased in this paper underwent 1.5 cycles of the UCD process and requires further testing to identify whether the challenges uncovered in the usability study have been resolved with the latest iteration of design and development work. Owing to the small sample size, the usability study was also not statistically powered. In this case, the evaluation of the results is mostly limited to qualitative data analysis. Although a questionnaire was completed by each user after the session, these results were only used to support the conclusions drawn from the qualitative data.

Finally, the participants may have had social desirability bias when answering interview questions and filling out the questionnaire, especially because they knew that the study coordinator also had involvement in the design and development of the voice app [37]. When things went wrong, the participants most often felt as though it was their fault for not understanding how to use the technology and, as a result, would not bring up comments regarding what they disliked about the experience.

### Conclusions

This project involved the design, development, and usability evaluation of a voice app for HF management. A UCD process was followed to systematically create a voice app that would meet user needs and be easy to use. The usability study performed at the PMCC at UHN provided insightful user feedback about the voice app’s design, with the overall response being positive with high user satisfaction. The findings from this usability study impacted the redevelopment of the voice app, which will be used in a future clinical evaluation. As advancements in VUIs progress, we believe they will play an integral role in providing access to chronic disease management programs by (1) helping more patients complete their tasks independently, (2) offering a more convenient experience to record relevant data, and (3) allowing those with limited technical and English skills access to these programs. The findings from this research show promise in using VUIs to help with chronic disease management.

## Appendix

Multimedia Appendix 1 Design requirements (including both user and software) that were considered during the design stage (Table S1). Software architecture diagram of the Medly voice app system (Figure S1). Instruction card provided to users to help them navigate the conversation (Figure S2). An example of the “script” describing the conversation for the Medly voice app (Figure S3). A “script” describing the conversation that will occur if the Medly voice app did not correctly capture the user’s symptom responses (Figure S4).

Multimedia Appendix 2 The System Usability Scale questionnaire that was handed out to the participants at the end of the usability session.

Multimedia Appendix 3 List of potential errors identified from usability study results, along with risk levels and mitigation strategies (Table S1). Main themes derived from the usability sessions (Table S2).

## Abbreviations

HF: heart failure
mHealth: mobile health
PMCC: Peter Munk Cardiac Centre
SUS: System Usability Scale
UCD: user-centered design
UHN: University Health Network
VUI: voice user interface

## Data Availability

The data sets generated and analyzed during this study are not publicly available due to an agreement with the Research Ethics Board of the University Health Network, which describes very specific circumstances that prohibits us from sharing the data on a public platform; however, the data are available from the corresponding author on reasonable request.
