# Supplementary material for: Design of a Patient Voice App Experience for Heart Failure Management: Usability Study
Source: JMIR Form Res. 2022 Dec 6;6(12):e41628. doi: 10.2196/41628 (PMC9768654; doi:10.2196/41628)
Supplement: Multimedia Appendix 1 [file formative_v6i12e41628_app1.docx]

**Multimedia Appendix 1**

**Table S1. Design requirements (including both user and software) that were considered during the design stage.**

|  | REQ. I.D. # | Requirement Description |
| --- | --- | --- |
|  |  |  |
| User Requirements |  |  |
|  | User Req 1.0 | The user shall be able to access the voice app on the Amazon Alexa device at any time, verbally. |
|  | User Req 2.0 | The user shall be able to verbally record their weight on the Medly voice app. |
|  | User Req 2.1 | The user shall be able to verbally record their blood pressure on the Medly voice app. |
|  | User Req 2.2 | The user shall be able to verbally record their heart rate on the Medly voice app. |
|  | User Req 3.0 | The user shall be able to verbally respond ‘yes’ or ‘no’ to the symptom questions asked by Medly voice app. |
|  | User Req 4.0 | The user shall be given the opportunity to correct any wrong measurements captured by the Medly voice app. |
| Software Requirements |  |  |
|  |  |  |
|  | SFW Req 1.0 | The Medly voice app shall give the user enough time to measure and record their weight and shall not time out. |
|  | SFW Req 1.1 | The Medly voice app shall give the user enough time to measure and record their blood pressure and shall not time out. |
|  | SFW Req 1.2 | The Medly voice app shall give the user enough time to measure and record their heart rate and shall not time out. |
|  | SFW Req 2.0 | The Medly voice app shall generate a response to the user, based on measurements inputted by the user. |
|  | SFW Req 3.0 | The Medly voice app shall send the data inputted and message it generated for the user to the clinical Medly dashboard to be viewed by the clinician. |
|  | SFW Req 4.0 | The Medly voice app shall confirm which measurements were captured with the user. |


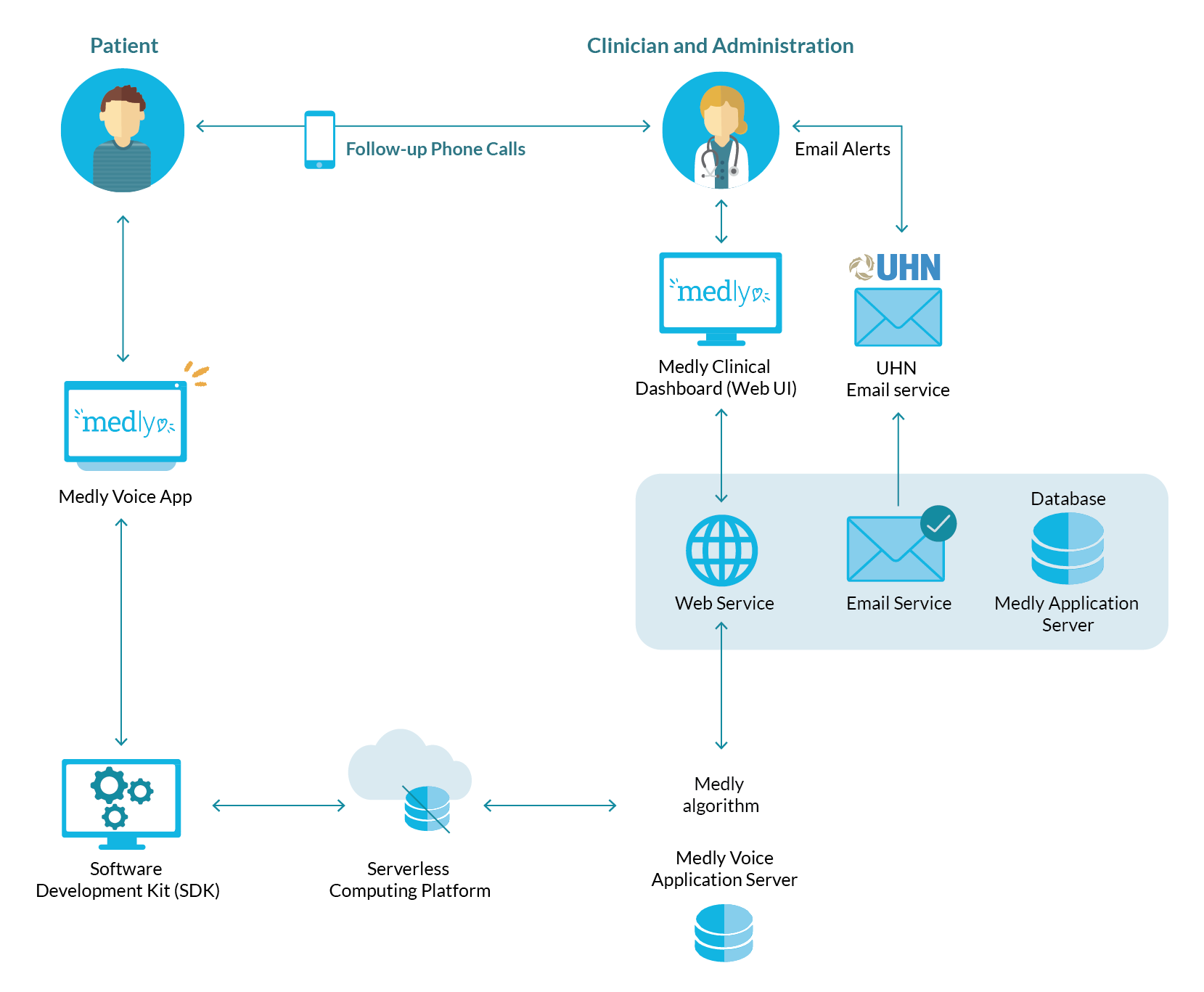


**Figure S1. Software architecture diagram of the Medly voice app system.**

*
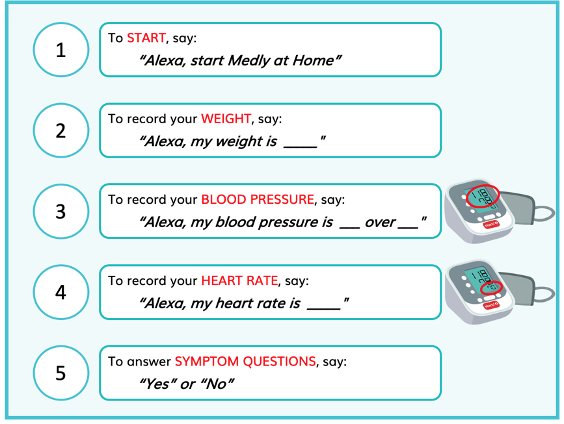
*

**Figure S2. Instruction card provided to users to help them navigate the conversation.**

*
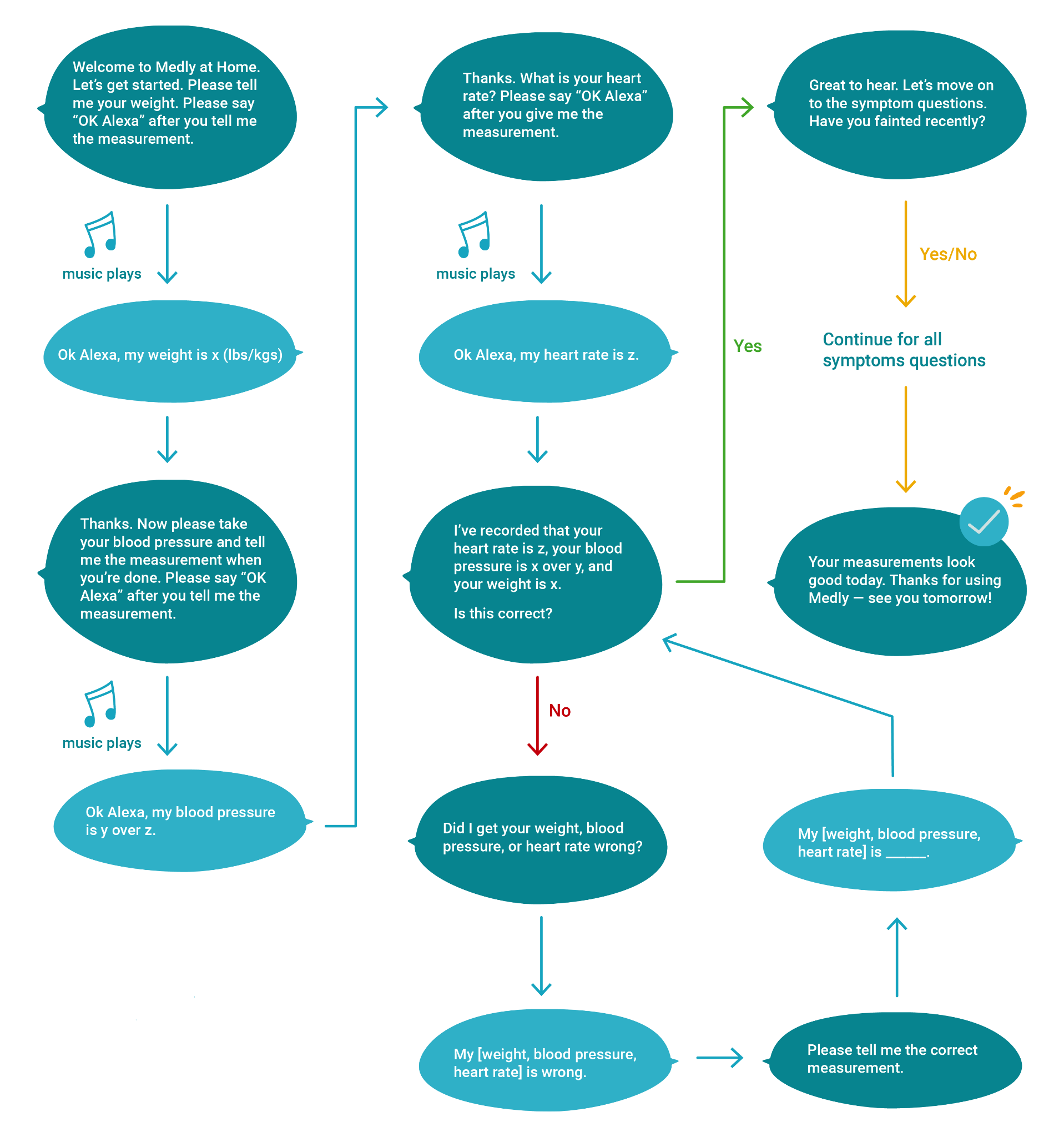
*

**Figure S3. An example of the “script” describing the conversation for the Medly voice app.**

*
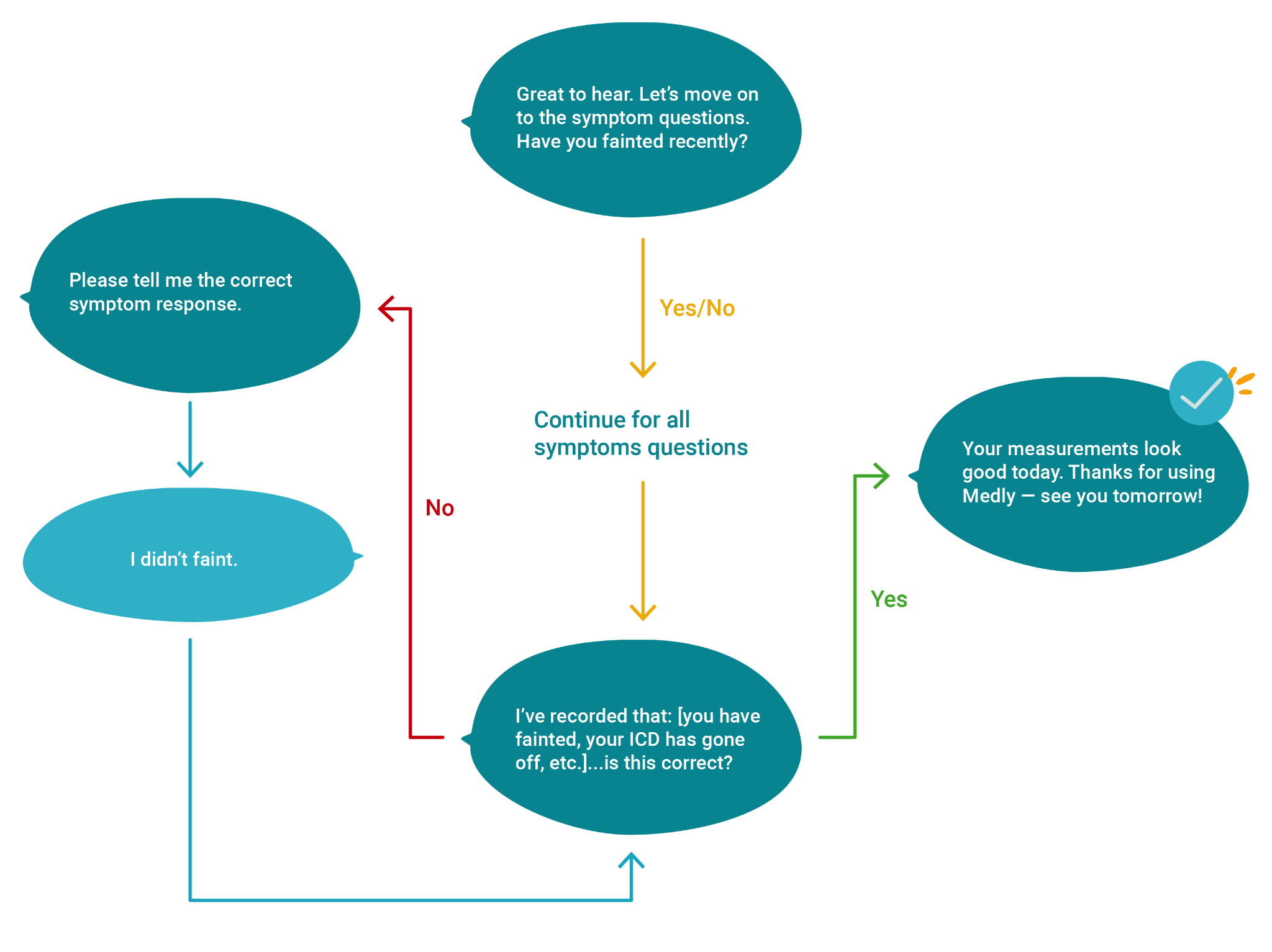
*

**Figure S4. A “script” describing the conversation that will occur if the Medly voice app did not correctly capture the user’s symptom responses.**
