# Supplementary material for: Design of a Patient Voice App Experience for Heart Failure Management: Usability Study
Source: JMIR Form Res. 2022 Dec 6;6(12):e41628. doi: 10.2196/41628 (PMC9768654; doi:10.2196/41628)
Supplement: Multimedia Appendix 2 [file formative_v6i12e41628_app2.docx]

**Multimedia Appendix 2**

| **Question** | **Strongly Disagree** |  |  |  | **Strongly Agree** |
| --- | --- | --- | --- | --- | --- |
| 1. I think that I would like to use this system frequently. |  |  |  |  |  |
|  | 1 |  |  |  | 5 |
| 2. I found the system unnecessarily complex. |  |  |  |  |  |
|  | 1 |  |  |  | 5 |
| 3. I thought the system was easy to use. |  |  |  |  |  |
|  | 1 |  |  |  | 5 |
| 4. I think that I would need the support of a technical person to be able to use this system. |  |  |  |  |  |
|  | 1 |  |  |  | 5 |
| 5. I found the various functions in this system were well integrated. |  |  |  |  |  |
|  | 1 |  |  |  | 5 |
| 6. I thought there was too much inconsistency in this system. |  |  |  |  |  |
|  | 1 |  |  |  | 5 |
|  |  |  |  |  |  |
| 7. I would imagine that most people would learn to use this system very quickly. |  |  |  |  |  |
|  | 1 |  |  |  | 5 |
| 8. I found the system very cumbersome to use. |  |  |  |  |  |
|  | 1 |  |  |  | 5 |
| 9. I felt very confident using the system. |  |  |  |  |  |
|  | 1 |  |  |  | 5 |
| 10. I needed to learn a lot of things before I could get going with this system. |  |  |  |  |  |
|  | 1 |  |  |  | 5 |

**Figure S1. The System Usability Scale questionnaire that was handed out to the participants at the end of the usability session.**
