# Supplementary material for: Design of a Patient Voice App Experience for Heart Failure Management: Usability Study
Source: JMIR Form Res. 2022 Dec 6;6(12):e41628. doi: 10.2196/41628 (PMC9768654; doi:10.2196/41628)
Supplement: Multimedia Appendix 3 [file formative_v6i12e41628_app3.docx]

**Multimedia Appendix 3**

**Table S1. List of potential errors identified from usability study results, along with risk levels and mitigation strategies.**

| Potential Error | Likelihood (5 point scale; 1: rare, 5: almost certain) | Consequence (5 point scale; 1: negligible, 5: catastrophic) | Risk | Mitigation Strategy |
| --- | --- | --- | --- | --- |
| User saying: weight, blood pressure, heart rate in incorrect format | 4 | 3 | 12 | Provide specific instructions in help manual describing how to say the values (100 instead of 1, 0, 0). Add “fallback phrases” (what Alexa says if it did not pick up a response) so that the user has more chances at correctly inputting their measurements. |
| Device picks up different value than what the user said | 3 | 5 | 15 | Confirm the user’s weight, blood pressure, and heart rate as well as symptom responses they have answered ‘yes’ to. Give the user an option to correct any incorrect measurements. |
| User not pausing enough before saying yes/no | 3 | 1 | 3 | Add fallback phrases to provide the user with more chances. |
| User taking too long to complete a measurement | 5 | 5 | 25 | Non-lyrical music implemented to prolong the wait period (~4mins) and re-prompt period (~3min period). |
| User only inputs measurement on Amazon Alexa and does not use smartphone. | 3 | 5 | 15 | Visual and verbal prompts during interaction to remind the user. Reminders also provided in instructions manual given to the user. |
| User wants to pause the app | 1 | 4 | 4 | Create an intent with utterances that the user may say to pause the app. If they say pause, Alexa informs them that pausing is not possible and that no measurements will be recorded. |
| User says something unexpected (clarifying question, etc.) | 3 | 3 | 9 | Brainstorm any clarifications the user may have while using the app and include part of the interaction model. |

**Table S2. Main themes derived from the usability sessions.**

| Theme | Theme Description |
| --- | --- |
| Changes in physical behaviour | Physical behaviour in most users who did not use a VUI before showcased nervous and tense feelings. These participants were very focused and constantly kept making eye contact with the study coordinator for confirmation, re-assurance etc. Participants who interacted with a device like this previously were more relaxed. |
| Preference between voice app and smartphone | When asked, most participants said they would prefer accessing *Medly* using the voice app over the smartphone. Common reasons include “the smartphone being frustrating to use”, “not needing to type in the numbers on their phone”, “not needing to turn the phone on to input the measurements on *Medly*”, and the voice app “being convenient and a bit easier than the smartphone”. |
| Importance of music during voice app interaction | Playing non-lyrical music while the users measured their weight, blood pressure, and heart rate served as a useful indicator that the device did not time out as they were performing their measurements and they did not feel rushed |
| Lack of privacy concerns | Very few concerns were brought up by participants relating to privacy when asked about general concerns they may have about the device. Comments included an uneasy feeling about the device recording conversations, and worry about sharing confidential information. |
| Desired reassurances during voice app interaction | Having the voice app re-iterate measurements was perceived as helpful by most of the participants since it gave them re-assurance that the data was correct and provided the confirmation that they were used to seeing on the *Medly* app. One participant noted it was more helpful hearing the measurements verbally, instead of visually, due to their dyslexia. |
| Helpful aids during voice app interaction | All users thought that the instructions card was helpful and would be required to help them navigate through the voice app (for at least the first few times). |
